# Community advisory boards as implementation strategies to center partner and patient voice in community health centers

**DOI:** 10.1017/cts.2024.679

**Published:** 2024-12-18

**Authors:** Rebekka M. Lee, Kamini Mallick, James G. Daly, Vetta Sanders Thompson, Elise Hoffman, Maria Papadopoulos, Stacey Curry

**Affiliations:** 1 Harvard T.H. Chan School of Public Health, Boston, MA, USA; 2 Washington University, St. Louis, MO, USA; 3 DotHouse Health, Boston, MA, USA; 4 Coastal Family Health Center, Biloxi, MS, USA

**Keywords:** Community health centers, implementation strategies, community advisory boards, engagement, mixed methods

## Abstract

**Introduction:**

Community advisory boards (CABs) are a promising approach for strengthening patient and partner voices in community health center (CHC) evidence-based decision-making. This paper aims to describe how CHCs used CABs during the COVID-19 pandemic to improve the reach of testing among populations experiencing health disparities and identify transferable lessons for future implementation.

**Methods:**

This mixed methods study integrates brief quantitative surveys of community engagement (*N* = 20) and one-on-one qualitative interviews (*N* = 13) of staff and community partners engaged in CHC CABs with a cost analysis and qualitative feedback from CHC staff participating in an online learning community (*N* = 17).

**Results:**

Community partners and staff engaged in the CHC CABs reported high ratings of engagement, with all mean ratings of community engagement principles above a 4 (“very good” or “often”) out of 5. Qualitative findings provided a more in-depth understanding of experiences serving on the CHC CAB and highlighted how engagement principles such as trust and mutual respect were reflected in CAB practices. We developed a CHC CAB toolkit with strategies for governance and prioritization, cost estimates to ensure sustainment, guidance on integrating quality improvement expertise, testimonies from community members on the benefits of joining, and template agendas and facilitator training to ensure meeting success.

**Conclusion:**

In alignment with the Translational Science Benefits Model, this study expands research impact through comprehensive mixed methods measurement of community engagement and by transforming findings into an action-orientated guide for CHCs to implement CABs to guide evidence-based decision-making for community and public health impact.

## Introduction

Community health centers (CHCs) are critical settings for addressing health disparities, sitting at the unique intersection of clinical care and daily life in communities experiencing the greatest health inequities. They offer comprehensive, patient-centered care for uninsured and underinsured populations through a primary care model. Over 90% of CHC patients are socioeconomically disadvantaged and 62% are people of color, two populations known to experience health disparities [1]. The potential population reach is tremendous – over 28 million people receive care at federally qualified health centers[1] and an even greater number are served by hospital-run health centers or live in surrounding neighborhoods where they could be positively impacted by CHC evidence-based practices and policies. CHCs excel at providing early detection, in part due to required clinical quality measures; however, primary prevention relies on behavioral interventions that cannot be conducted in a single office visit. Implementation science has helped advance the adoption of evidence-based interventions (EBIs) within the clinical walls of CHCs, and community partnerships have been held up as a promising approach for addressing health equity. However, limited research has focused on *how* CHCs can successfully collaborate with community partners to increase their impact on primary prevention.

The Translational Science Benefits Model (TSBM) seeks to expand documentation of research impacts beyond traditional research metrics (e.g. cost effectiveness, health care accessibility, public health practices) [2]. In this paper, we focus on community impacts within implementation science – presenting a mixed methods approach for ensuring comprehensive assessment of engagement among community implementation partners and an example of how data can be integrated to create an action-oriented implementation strategy designed by and for community partners.

In a recent pilot study, our team surveyed CHC staff in Massachusetts to document the extent to which they implement cancer prevention EBIs – specifically targeting nutrition, physical activity, and tobacco use – and used interviews to further explore the use of community partnerships to extend capacity [3]. We found a striking absence of community partnerships for implementation of cancer prevention EBIs [3]. At the same time, we had significant success helping Massachusetts CHCs develop local community advisory boards (CABs) to guide community engagement and strengthen partnerships for implementation of COVID-19 testing, leading to improved reach and participation among populations experiencing health disparities [4]. This study underscored the importance of community members as collaborators and identified a great need for a resource to help CHCs develop and implement a structure to ensure high-quality engagement. In the current study, we report on research we conducted that employed validated surveys and qualitative interviews to assess engagement among CAB members as well as CHC staff who attended CAB meetings. Pairing this mixed methods approach with a cost analysis and CHC feedback, we developed an implementation strategy in the form of a practical toolkit designed for CHCs to plan and implement CABs to guide evidence-based decision-making.

## Methods

### Setting and design

The Implementation Science Center for Cancer Control Equity (ISCCCE) is a research center funded by the National Cancer Institute. A collaboration between the Massachusetts League of Community Health Centers (Mass League), the Harvard T.H. Chan School of Public Health, Massachusetts General Hospital, and Dana Farber Cancer Institute, the center supports implementation of research pilots based in CHCs [5]. During the height of the COVID-19 pandemic, ISCCCE received a competitive supplement from the RADx-UP program to improve testing in collaboration with CHCs in communities with high rates of disease and racial/ethnic disparities [4]. One aspect of our RADx-UP approach was to require local CABs to inform tailored delivery and communications to promote COVID-19 testing. Researchers provided all CHCs suggested guidance on composition (e.g., include 2+ patient representatives and local board of health member), staffing for administration, and ideas for the first agenda. However, each CHC had the autonomy to set up its own governance structure, membership, and meeting cadence. A member of the Mass League team provided technical assistance for developing the CABs. The individuals in this study were actively engaged in CHC-based CABs in 2021–2022 and participated in retrospective surveys and interviews in Spring/Summer 2023.

**Figure 1.**
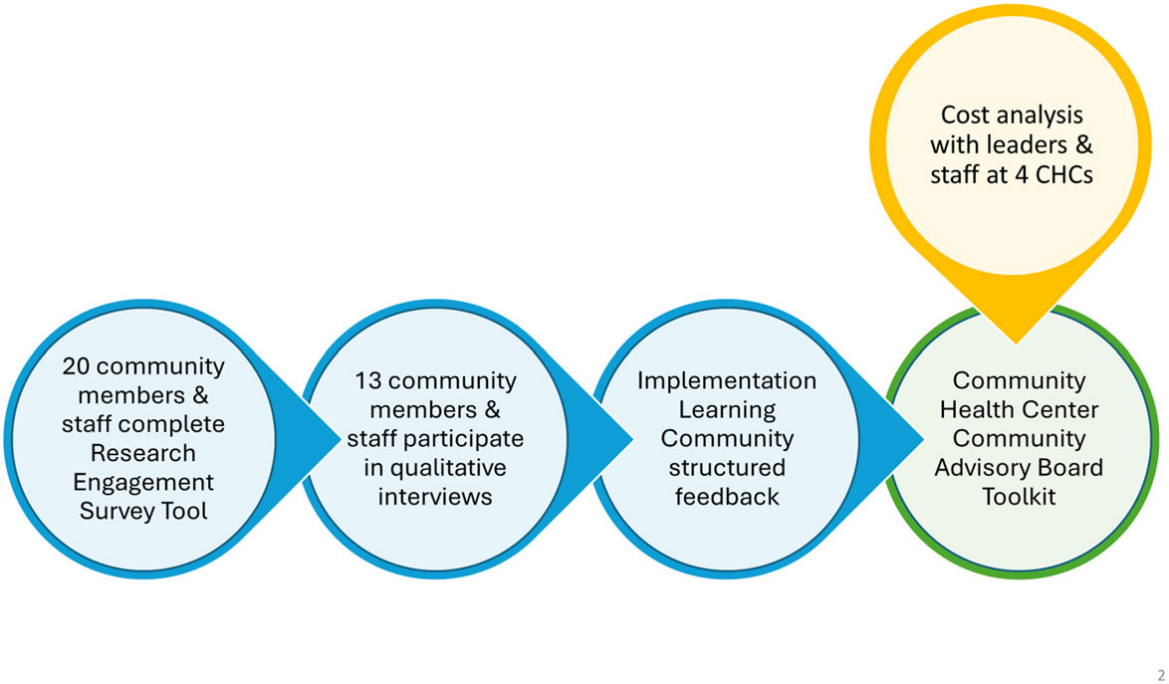
Mixed methods design.

This study uses a mixed methods design to integrate quantitative and qualitative data from community partners and CHC staff to build an implementation strategy – a toolkit designed for CHCs to plan and implement CABs (Figure 1). The study was approved by the Harvard Longwood Campus Office of Regulatory Affairs and Research Compliance.

### Quantitative surveys

Researchers invited all community partners engaged by CHC-led CABs and CHC staff who regularly attended CAB meetings to complete a 5-minute online survey via the REDCap platform. CHC leaders sent email notifications to their CAB members to share information about the study several days prior to when formal recruitment began. The study staff sent an email invitation to complete the survey through REDCap. The Research Engagement Survey Tool (REST) addresses nine focal areas aligned with core principles in the community engagement literature (see Table 1) [6]. The brief version used in this study included nine close-ended items on the quality and quantity of each partner’s engagement experience and background information about themselves (e.g., demographics, expertise) [7]. The brief REST has high internal consistency (Cronbach’s alpha = 0.92 for quantity scale; 0.94 for quality scale) and is significantly correlated with the comprehensive (32-item) version of the tool (*ρ* = 0.97; *p* < 0.001 for both scales) [7]. Items were adapted slightly to capture the relationship between community partners and a CHC instead of researchers, which the tool was originally designed to measure. Respondents were compensated $50 for survey participation. We used descriptive analyses to summarize CHC responses and variability across CHC and by role.

**Table 1.** Research engagement survey tool results among 20 members of 4 community health center community advisory boards

Engagement Principle	Survey item	Quality Rating Mean (Standard Deviation) Poor 1 - Excellent 5	Quantity Rating Mean (Standard Deviation) Never 1 - Always 5
Focus on community perspectives and determinants of health	The focus is on problems important to the community	4.60 (0.60)	4.60 (0.50)
Partner input is vital	All partners assist in establishing roles and related responsibilities for the partnership	4.35 (0.88)	4.40 (0.82)
Partnership sustainability to meet goals and objectives	Community-engaged activities are continued until the goals (as agreed upon by all partners) are achieved	4.35 (0.75)	4.55 (0.51)
Foster co-learning, capacity building, and co-benefit for all partners	The partnership adds value to the work of all partners	4.70 (0.47)	4.55 (0.51)
Build on strengths and resources within the community	The team builds on strengths and resources within the community	4.65 (0.49)	4.55 (0.51)
Facilitate collaborative, equitable partnerships	All partners’ ideas are treated with openness and respect	4.95 (0.22)	4.85 (0.37)
	All partners agree on the timeline for making shared decisions about the project	4.26 (0.81)	4.35 (0.67)
Build and maintain trust in the partnership	The partnership’s processes support trust among all partners	4.60 (0.60)	4.70 (0.47)
	Mutual respect exists among all partners	4.85 (0.50)	4.85 (0.37)

### Qualitative interviews

Participants who completed the survey were invited to participate in semi-structured interviews over Zoom. The interview guide was structured to gain a more in-depth understanding of CAB members’ experiences on CHC-led CABs and to support the development of a CHC CAB toolkit. The interview guide included questions on strengths and challenges of the CAB, greatest accomplishments, and recommendations for CHC leaders who are interested in starting a CAB. Probes in the interviews also aligned with the REST items. For example, to follow-up on the engagement principle of respect, interviewers asked participants to share specific examples of when they felt respected and why they felt they could trust their partners. See Supplemental Materials for full interview guide. Two research staff members conducted most of the interviews together; one served as the primary interviewer and the other took notes and asked follow-up clarifying questions as needed. Participants received $75 for interview participation. All interviews were audio-recorded and transcribed.

Interviews were analyzed using the qualitative analysis software, NVivo. First, interviews were coded deductively based on CHC CAB toolkit sections pre-determined by the study team (e.g., Leadership, Meeting Facilitation, Prioritization). Next, interviews were inductively coded to capture the experience and engagement of participants. Examples of inductive codes included Motivation to Join, Value of CAB, Impact on Self, and Impact on Community. Thirty percent of interviews were double-coded to ensure reliability and multiple perspectives. All discrepancies were resolved through consensus discussion.

### Cost analysis

CHC leaders were interviewed to conduct a cost analysis of the resources needed to implement their CABs. We used a budgetary costs perspective to determine all costs associated with CABs implemented at the CHCs. Data were collected via a series of two meetings in 2023 with each CHC to retrospectively determine costs associated with implementing CABs in 2021–2022. During the meetings, the study team used a standardized template for costing to guide a structured, collaborative conversation with CHC staff. We began by identifying all activities needed to plan, coordinate, and implement the intervention, such as recruitment of CAB members, meetings, trainings, and communications. Next, we identified resources associated with each activity, such as paid labor, travel, consumable materials, and equipment. We also measured the amount of those resources needed (e.g., hours of staff time, number of gift cards purchased). Finally, we assigned monetary value to each of the resources. Salaries were estimated using state-level data from the National Association of Community Health Centers and a 28% fringe rate as reported by the Mass League [8]. No costs related to research and reporting were included in the cost estimate. Once costs were collected, they were categorized by phase (e.g., planning vs. implementation) and resource type (e.g., paid labor, incentives).

### Structured feedback

In June 2023, we gathered structured feedback from CHC staff during a recorded two-hour Implementation Learning Community (ILC) Meeting over Zoom. We began by introducing the purpose of the toolkit and then used a series of collaborative activities to gather qualitative input. The PI and study staff facilitated the meeting with structured prompts to invite constructive feedback and broad participation, dividing participants into breakout groups by their level of experience with CABs (none, some, or a lot). Discussions began with broad questions on potential benefits and challenges of implementing CHC-led CABs and what participants would find helpful in a toolkit. Next, facilitators asked participants specific questions to gather feedback on topics identified as key gaps: leadership structure, prioritization, and sustainability. Feedback was captured verbally via recording and through an interactive, online whiteboard.

### Toolkit development

The research team integrated data from surveys, interviews, structured group feedback, cost analyses, and best practices identified from the literature to develop a CHC-based CAB implementation strategy in the form of a practical “how to” toolkit. We reviewed eight existing resources, including toolkits written to guide researchers in creating CABs, a community-based participatory research curriculum, and patient engagement guidance [9–16]. Based on best practices from reviewed materials and consultation with the study team, we identified seven sections to structure the toolkit: CAB Leadership structure, CAB Member Recruitment, Meeting Logistics, Meeting Facilitation, Prioritization, Cost & Sustainability, and Evaluation. To center the voices and experiences of CAB members, these best practices were combined with findings from the structured feedback session and qualitative interviews. Cost data was integrated to assist with budgetary and cost-sharing planning, a major priority expressed by the Mass League. The study team tracked which data sources contributed to each toolkit addition. The toolkit is available in Supplemental Materials and on our website [17].

## Results

### Participants

Of 26 people invited, 20 people from four CHC CABs completed the survey (77% response rate). Fifteen were women and five were men. When asked to select their role (multiple options allowed)participants identified as community-based organization partners (*N* = 6), community residents (*N* = 6), government partners (*N* = 2), and patients of the CHC (*N* = 4). Ten CHC staff who regularly attended CAB meetings completed the survey. Approximately 40% of participants identified as White, 25% as Black, 25% as Hispanic/Latino, 10% as Asian, and 10% as “other” race. Respondents’ ages ranged from 24 to 67 (mean 47). About 70% of participants reported they had some experience working with the CHC prior to joining the CAB; 20% said this prior experience was as part of a formal advisory or governing group and 15% said they had no prior experience working with their CHC. Thirteen people who completed the survey agreed to participate in follow-up qualitative interviews. We interviewed at least one staff member and one community member from each of the four CHCs represented in this study.

### Quantitative surveys

Participants reported high “quality” (e.g., how *well* partners followed each principle) and “quantity” (e.g., how *often* partners followed each principle), with all mean ratings above a 4 (“very good” or “often”) (Table 1). Ratings were highest for principles focused on trust. For example, the item “all partners’ ideas are treated with openness and respect” was rated an average of 4.95 for quality with almost all “excellent” ratings and 4.85 for quantity with almost all “always” ratings. More task-oriented items focused on establishing roles, agreeing on timelines, and meeting goals had mean ratings between 4.25 and 4.55, indicating positive, but more mixed experiences. Staff ratings were slightly higher than those of CAB members for quality ratings (average 4.61 vs. 4.57), but lower for quantity ratings (average 4.47 vs. 4.73).

### Qualitative interviews

Findings are presented according to deductive codes corresponding with the sections of the CHC-based CAB Toolkit and key inductive findings that emerged.

#### Starting a CAB: recruitment, motivation, and expectations

CHC leaders recruited both community members and staff to join CAB meetings. Most community members were those with whom leaders were already familiar, though a few were identified through professional networks. The motivation for almost all interviewees to join the CAB was alignment with the topic of focus - to help address COVID-19 challenges. Most were already involved in local response efforts before joining and saw the CAB as an opportunity to support the CHC to better reach community members and to learn from one another’s experiences and share resources. One community partner shared, “We needed whatever resources and partnership we could get. You know, there was more to be done that we could achieve alone.”

CHC staff typically joined as part of their job duties and served as a link between CAB members and CHC leadership. Being told, rather than asked, to join meetings affected one staff member’s initial experience engaging with the CAB. Unclear expectations presented a challenge; they felt unable to fully participate in initial meetings because they were told to join with little instruction and felt intimidated by others’ experience and rank. Interviewees had varying ideas of what their roles and responsibilities were at the start: some borrowed from previous experiences on advisory boards to shape their expectations, while others said they gained a better understanding over the course of several meetings.

For future CAB recruitment, interviewees strongly recommended articulating a clear CAB purpose, expectations, and time commitment when recruiting members. All interviewees emphasized the importance of identifying a diverse group of individuals that represent the surrounding community, addressing racial and ethnic diversity, as well as differences in socioeconomic backgrounds, genders, ages, cultures, and occupations. One community partner shared:

And by diversity, I’m not just saying cultural diversity… I think there’s value in having leaders from organizations as well as direct service providers as well as community members, maybe faith-based [representatives]. What are some of the nontraditional people that aren’t usually part of [a CAB]?

A couple of participants recommended recruiting individuals who are enthusiastic about community work and willing to put in the time and effort to serve on the CAB, reinforcing the need for sharing expectations during recruitment. While it may be intuitive to reach out to outgoing people, one person cautioned against overlooking quieter people saying, “Sometimes you don’t know how powerful your voice is until you’re given the option to have it be powerful.” Interviewees suggested CHC leaders should start by asking schools, hospitals, and local faith groups for suggested CAB members. One interviewee recommended asking patient-facing staff, like nurses, medical assistants, and providers because they interact with community members frequently and “know who is going to speak up.” Another participant suggested starting the search for potential members with those who are already part of other groups and coalitions with a similar purpose to not duplicate efforts and learn who in the community may be aligned with the proposed CAB’s mission.

#### Running CAB meetings

##### Meeting logistics

Interviewees reported a successful CAB meeting involved having a clear and flexible agenda that allows for natural discussion, good facilitation and time management skills, and consistent communication after meetings to implement recommendations and follow-up on questions raised in the meeting. Interviewees recommended sending agendas before the meeting and notes afterward to ensure people can follow progress and have clear expectations for the next meeting, especially to prioritize their attendance.

Scheduling and consistently attending CAB meetings was a major challenge throughout all interviews. While some people were able to prioritize attending every meeting, others said they could not always do so due to competing responsibilities. Inconsistent meeting attendance sometimes affected the flow of the CAB. Ideas shared in one meeting could not be built upon in the next. One member shared:

Someone would be there, and they’d come up with this great idea. We’d be like, “All right. Well, how can we help you?” And maybe, “Okay, you can do this, this, and this” And then we wouldn’t see them again for a while.

Failing to attend a meeting influenced some others by not prioritizing future meetings.

##### Leadership

Most CABs were led by CHC staff, but they varied in structure (Table 2). Participant impressions of the leadership were positive. When asked how well leadership followed through on CAB input, most thought leaders were very intentional about translating ideas and feedback into practice. For example, the leader at one CHC implemented new working hours for the community van at the insistence of the CAB to improve availability in the community. One CAB member reported it was evident how much leaders valued the CAB by prioritizing attending meetings:

But her presence is telling. Not only that she wants to move with this, but she wants to put this group on the highest of her priorities. She participated in every single meeting. That alone was telling. To be there alone was telling. Somebody in her capacity would find an hour or an hour and half every week to meet with this group. That shows you how important this thing is for them.

**Table 2.** Integrating quantitative survey, qualitative interview, and implementation learning community structured feedback data to build community health center community advisory board toolkit

Toolkit section	Data sources	
Leadership structure	Interviews & implementation learning community	Attendees shared that rotating board chairs helped to prevent burnout, leading to guidance on rotating chairs as an option for “Choosing a Chair” in the toolkit.
Member recruitment	Interviews	Interviewees discussed the importance of clearly communicating the purpose of the community advisory board and expectations of the member during recruitment. This feedback led to inclusion of exemplar quotes from interviews in the toolkit to demonstrate the importance of leading with the person’s value of joining and ensured that the new members are aware of the expectations.
Logistics	Interviews & Survey	Interviewees shared strategies that made meetings work more effectively, including sending reminders, clear agendas, and meeting minutes. These steps were included in a planning checklist in the toolkit.
Meeting facilitation	Interviews & Survey	Survey ratings of trust and respect were high. Interviews reveal that leaders who skillfully “tapped in” quieter members supported engagement. This tip was shared as a best practice for meeting facilitation in the toolkit.
Prioritization	Implementation learning community & Survey	Meeting attendees discussed previous experiences using decision matrices, such as impact vs. implementation[18], as helpful strategies. This tool was included in the toolkit with an example Appendix H.
Cost and sustainability	Costing & implementation learning community	Costing interviews provided estimates of the cost of running community advisory boards at four health centers. These costs, broken down by category, were included in the toolkit.
Evaluation	Survey & interviews	Interviewees suggested that members be surveyed or interviewed to understand their experiences serving as community advisory board members. This led to the inclusion of example interview questions and a validated survey leaders could use to ask members about their first year of membership.

##### Meeting facilitation

Participants reported that CHC leaders ran the first few CAB meetings and then passed on or shared meeting facilitation to another staff member. Participants felt like they were trusted and valued members of the group. When prompted about when they felt that sense of respect and trust, many said they could feel it right from the beginning and attributed it to the shared goals of the group. One interviewee shared, “It took us two weeks after the initial start to really form that bond and realize that this is a very strong group and we can mobilize people. We can do something to help our community because that was the main goal.”

Meeting facilitators were an important part of the success of these CABs as they helped to set the tone of meetings, guided discussion, and ensured every voice was heard. The staff member who initially felt unsure of their role in the CAB shared that she started to feel like she belonged because facilitators made intentional time for her opinions, “They started asking me, “What do you think? How do you feel about this?”… [I]t was more inclusive.”

Facilitators who balanced the importance of different ideas and topics contributed to building trust:

I may have been part of the CAB because my focus was like – I really want to see the homeless population served. Somebody else was really focused on maybe the minority population. It never felt like one was more important than the other, or that one person’s primary focus got more attention than the other like it. It really was very well-rounded and kind of holistic that everybody got to kind of put their opinions and priorities forward.

A further sign of respect was good time management – running the CABs on time and ending on time. The facilitators built open dialog and flexibility in the agenda to discuss different topics but also kept the CAB focused. A member shared, “The leadership was really superb. It was like every meeting there was like a focus. There was an agenda. There was a parking lot, for you know, things that we had to bring up from a previous meeting.” This way all ideas were heard, but were prioritized to ensure meetings could be productive and remain in the timeframe asked of CAB members. Respect was also built through the willingness of CAB members themselves to be open to discussion, take turns to speak, and try new things. One interviewee shared:

There was that respect. And from that respect, you built that trust – we all had that open mind to listen to one another and try new things. …Sometimes you go into some of these groups and they already have their agenda and it may feel like they are just checking off the boxes. But in this case, it didn’t feel like that. They would give you a suggestion and then it would be like, “Oh I didn’t think about it like that.” And they would be like, “Okay, let’s try it.”

For future CABs, interviewees recommended choosing facilitators who can create a welcoming environment where all voices are valued and given equal importance. For those who are less vocal, participants suggested reaching out ahead of time to see how they would best like to be engaged.

#### Value of the CABs

Most participants shared that the greatest value of the CAB was its positive impact on the community. For example, CHCs were able to expand their reach to many more people within their catchment areas because of the different outreach and communication strategies suggested, including diversifying outreach locations and connecting with more community-based organizations. An interviewee said:

I think that they really allowed us to nail where the access issues were and how best to reach those patients that we wouldn’t otherwise be able to reach. …Ideas emerged from these CABs that we implemented and were a huge part of the reasons for some of the successes we had.

Only one participant shared that the CHC CAB did not add value to their work. This person was already heavily involved in COVID-19 relief efforts within their city when the CAB was formed, so they felt their role on the CAB was only to provide updates on their city’s pandemic strategies. For this member, CAB-related efforts felt redundant, but they acknowledged the value of the CAB to people from nearby communities who were just starting with their COVID-19 response. For example, another member of this CAB felt “overwhelmed with gratitude” for participating. They did not feel as siloed in their COVID-19 work as they did before joining and thought it was helpful to learn about successful strategies and resources. This CAB member said, “I would not have been able to do that as me, as by myself. There’s no way… I wouldn’t have had the support. So, having a community focus for giving you the ideas. Fresh ideas. Recommendations. Just being there supportive made a big difference.”

Participating in a CHC CAB increased some members’ professional network, which enabled them to call on their new relationships for help on other community projects or issues. For example, one participant mentioned relying on their new connections to help a community member transfer school medical records. Finally, two staff members indicated serving on the CAB led to personal development, saying it made them feel more confident sharing their thoughts and speaking up regardless of who is in the room and feeling intimidated. One interviewee summed up this sentiment:

[I learned] to have more confidence in myself; and also in the confidence of my team and the people that I work with, because obviously I didn’t quite understand why I was there. But there was a reason, and they had faith in me that I need to be there, and I was the right person to be in that group…When I’m in any situation or in any meeting or anything that I don’t quite understand why I’m there, I now speak up.

### Cost analysis

**Figure 2.**
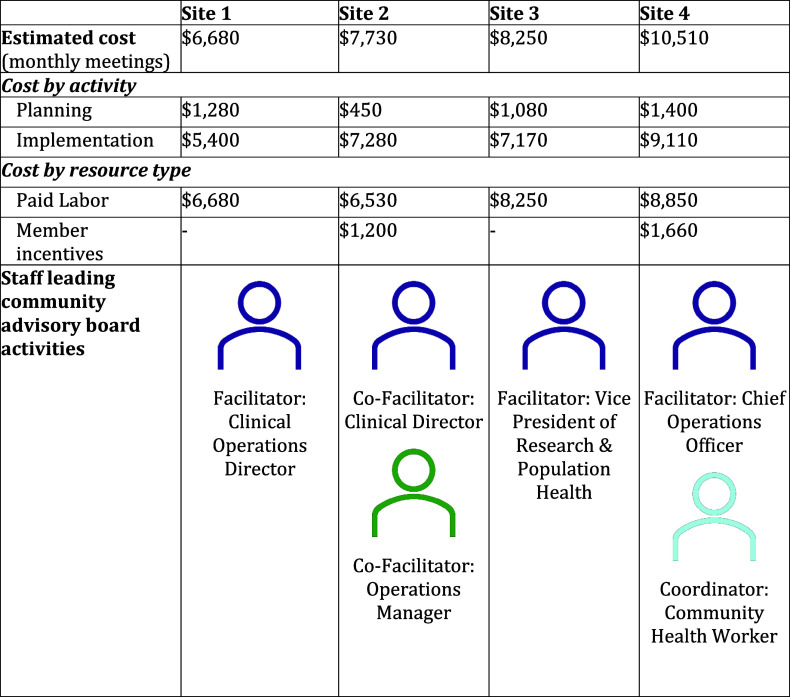
First year cost estimates for community health center community advisory boards.

The estimated annual cost for implementing a CHC-based CAB was $8,000, ranging from $6,680 to $10,510 across the four sites (Figure 2). Paid labor of existing staff was the largest type of cost, accounting for between 84% and 100% of cost estimates. Two CHCs offered incentives to members in the form of gift cards ($25–$50 per meeting) or stipends after completion of the project. Given the urgency of the pandemic, these CABs chose to meet monthly. However, with a quarterly meeting structure, CAB would likely cost under $5,000 annually – estimates ranged from $2,880 to $4,390.

### Structured feedback

Seventeen attendees at the ILC meeting gave structured feedback on 3 main areas: 1) Leadership and Structure, 2) Prioritization, and 3) Funding and Sustainability.

#### Leadership and structure

Attendees shared examples of successful leadership structures from their previous experiences on CABs, quality improvement groups, and boards that included community members and staff leaders. These groups often did not have CAB chairs and when they did, chairs were frequently “voluntold.” Rotating chairs reportedly helped to share the load of responsibility and prevent burnout. Attendees discussed the importance of balancing the formality of the CAB with maintaining flexibility. CABs with more structure brought a sense of importance, while a less structured CAB was more accessible for community members to engage. Attendees discussed how CABs could function as a steering committee with several topic-focused groups reporting to them. Lastly, attendees suggested appointing one member as a liaison between the CAB and CHC leadership to effectively share ideas and enact change.

#### Prioritization

When asked about past experiences with prioritization, attendees noted that available funding and links to quality improvement metrics or other regulatory requirements were key considerations. While some attendees described using specific prioritization strategies (e.g., decision matrices), others shared the prioritization process worked best when decisions involved broad input and were driven by population health data or community health assessments. They suggested outlining clear guidance and examples on the use of prioritization strategies, including who should be involved in the decisions (e.g., leadership, patients), in the toolkit.

#### Funding and sustainability

Attendees discussed several potential sources to sustainably fund CABs, including grants, CHC operating budget, Accountable Care Organizations (ACOs), or value-based care contracts. Attendees noted funding should cover costs of Zoom, translation services, staff time, payments for members, data pulls, and dissemination. They requested the toolkit include simple templates for budgeting and suggested payments for CAB members.

### Toolkit development

Results of the mixed methods integration are displayed in Table 2 – they highlight how research findings were translated into actionable tips and resources for CHC staff.

## Discussion

This mixed methods study provides an in-depth understanding of what successful CHC CAB engagement looks like and a guide for future implementation. Results from the REST showed high levels of community engagement across all principles [6]. When paired with qualitative interviews, we discovered insights into actionable challenges such as defining roles early and structuring meetings to ensure follow-through. Interviews emphasized the importance of having members with a range of experience, aligning outreach messaging and implementation with members’ personal and professional priorities, and communicating with transparency to ensure trust. In contrast to traditional research CABs, many CHCs set up these groups to include community partners and residents as well as CHC staff who were members of the community and could speak to the feasibility of implementing strategies recommended. CABs had a positive impact on the community, expanding the reach of health services to diverse populations through partner-led efforts. Given these positive findings, we see great potential for CHC-based CABs to support implementation of EBIs across many topic areas, including cancer prevention; they may be particularly helpful for engaging partners to improve the equitable reach of prevention and care.

Integration of the quantitative and qualitative data into a CHC CAB toolkit serves as a public good of direct relevance to practitioners with the potential for community and public health benefit beyond typical research metrics – an exemplar of the TSBM [2]. It is designed to support CHCs in providing evidence-based community health services, disease prevention and reduction, and health education resources [2]. The toolkit is a valuable resource for CHC leaders who are new to setting up a CAB, providing practical “how to” guidance on setting up leadership structures, recruiting and engaging busy members, and facilitating community-focused meetings. These findings align with the research literature and serve to sharpen the focus on community-centered implementation science – identifying CHC-led CABs as concrete implementation strategies to be used to center community voice in the translation of evidence into practice [19–21]. As a next step, we will be studying the implementation of the CHC CAB toolkit paired with training and facilitation among six CHCs engaged in the newly funded Massachusetts Partnership for Community-Engaged Cancer Control Equity, with the aim of ultimately adapting and scaling statewide with our partners at the Mass League. Future research could add analysis of CAB meeting agendas and notes, using the TSBM impact tracker, to document outcomes within each category [2].

The context of this study highlights the importance of understanding the role and functioning of CABs in both emergency situations when there is an urgent need to address problems (e.g., pandemic outreach) and non-urgent circumstances (e.g., community input on chronic disease prevention priorities). CABs were assembled very quickly and run via monthly meetings to address rapidly shifting needs of the pandemic. Time dedicated to these CABs was likely more than typical, but other costs (e.g., parking and food for in-person meetings) might be higher in a non-emergency context. As CHCs develop CABs, they should include sufficient personnel time for planning and implementing meetings at least quarterly, standard compensation for CAB members, and identify sustainable funding streams to maintain CHC CABs outside of grant funding.

This pilot has limited generalizability given its focus on four CHC CABs. However, most of the toolkit domains have relevance across other practice settings and structured feedback during the ILC provided additional insights from many more CHC staff. We recommend potential users apply a similar approach to gather feedback on setting specific adaptations prior to implementation in other practice settings. The REST was not administered before or in the early phase of the CHC CAB process, while many of the experiences that were discussed as problematic in interviews were early on. The survey responses likely reflect overall feelings once the group had established some norms and expectations.

Both the measures collected and products developed via this study demonstrate the importance of thinking beyond traditional research metrics [2]. We recommend mixed methods, pairing survey measures like REST[6] with qualitative assessment, to ensure a comprehensive understanding of community benefits within research [22]. Finally, the CHC CAB toolkit serves as a public good of direct relevance to practitioners with the potential for community impact.

## Supporting information

Lee et al. supplementary material 1 Lee et al. supplementary material

Lee et al. supplementary material 2 Lee et al. supplementary material

## Supplementary material

The supplementary material for this article can be found at https://doi.org/10.1017/cts.2024.679.
