# Supplementary material for: Community advisory boards as implementation strategies to center partner and patient voice in community health centers
Source: J Clin Transl Sci. 2024 Dec 18;9(1):e14. doi: 10.1017/cts.2024.679 (PMC11795859; doi:10.1017/cts.2024.679)
Supplement: Lee et al. supplementary material 1 — Lee et al. supplementary material [file S2059866124006794sup001.pdf]

Community Health Center Community Advisory Board Toolkit

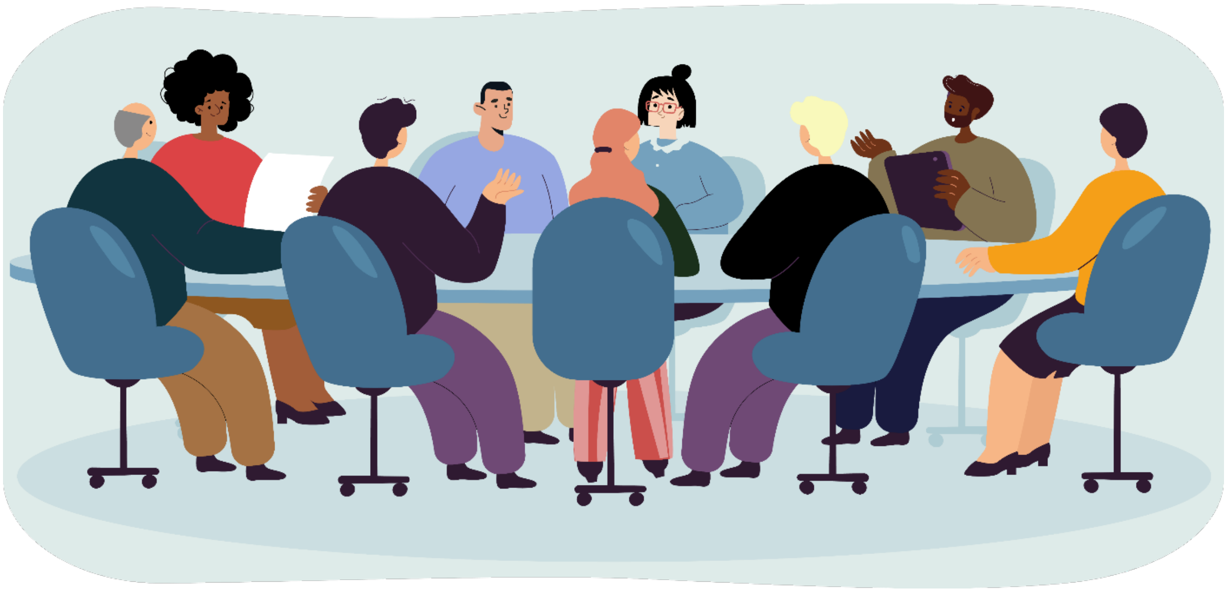

The authors acknowledge and appreciate the efforts of all participating CHCs and CAB members who contributed to its content. This toolkit was made possible with help from the Implementation Science Center for Cancer Control Equity, a National Cancer Institute funded program (P50 CA244433). The content is solely the responsibility of the authors and does not necessarily represent the official views of the National Cancer Institute.

ISCCCE
IMPLEMENTATION SCIENCE CENTER FOR CANCER CONTROL EQUITY

Table of Contents

Introduction **3**

CAB Leadership Structure **4**

Considering formal and informal structures 4

CAB Leadership Structures 5

Choosing a Chair 6

Key Takeaways 7

CAB Member Recruitment **8**

Identifying Members 9

Recruiting Members 10

Characteristics of Effective CAB Members 10

Member Agreements 11

Member Onboarding 11

Key Takeaways 11

Meeting Logistics **12**

Planning Checklist 12

Key Takeaways 12

Meeting Facilitation **13**

Best Practices 13

Key Takeaways 13

Prioritization **14**

Why use a prioritization strategy? 14

Before Prioritization 14

Prioritization Strategies 15

Key Takeaways 15

Cost and Sustainability **16**

Budgeting for Staff Time 16

Additional Items for your CAB Budget 16

Case Example – RADx MA 16

Key Takeaways 18

Evaluation **19**

Surveys 19

Interviews 19

Key Takeaways 19

Appendices **20**

Resources **34**

Introduction

This Toolkit provides guidance for community health centers seeking to establish community advisory boards (CABs). A CAB is a group of community members and organizational representatives that provide guidance to a project or initiative. CABs have commonly been used in research, but could also be valuable structures to support health centers set priorities, implement evidence-based prevention and care, and improve outreach to residents. This guide combines established best practices with practical tips from health center staff, stories from health centers' successful experiences, testimonials from community members who have been part of CABs, and resources to support implementation. Health center leaders will find guidance on how to establish the leadership structure, plan the logistics and facilitation of meetings, prioritize initiatives, budget for sustained impact, and evaluate engagement. Overall, this toolkit provides a comprehensive guide for creating a well-structured and effective CAB, ensuring that it can make a meaningful impact on health in the community it serves.

CAB Leadership Structure

Establishing a structure will enable CABs to work more effectively together and ensure progress is made on their stated goals. CAB structures ensure that members and staff all have clear understandings of roles. Regardless of what structure is selected, all CAB members should have a shared understanding of the approach to ***communication***, ***decision-making***, ***prioritization***, and ***conflict resolution***. For each of those domains, the approach should be openly discussed and written down. In this chapter, you will have the opportunity to weigh the considerations of different leadership structures to determine the best fit for your CAB.

Considering formal and informal structures

When setting up a CAB, leadership structure and the level of formality are key considerations.

- The level of formality you choose for your CAB should align with the goals and purpose of your CAB.
- Consider what leadership structures are used in the CHC for other purposes and how they may be adapted for the CAB
- Consider the *experience* and *strengths* of the CAB members, the work you hope to achieve, and characteristics of the community
- Choosing a formal CAB structure does not necessarily mean that communication, decision-making, prioritization, and conflict resolution methods are built into the structure. While a formal structure may make it easier to establish these ground rules, this step should not be skipped.
    - For communication strategies see **Member Onboarding** and **Meeting Facilitation**
    - For prioritization and decision-making strategies see **Prioritization**
    - For conflict resolution strategies, see Appendix A for Community Tool Box Training on Conflict resolution

Formal Structure- A CAB with a named leader and defined meeting rules for approving agenda items and making decisions. It includes2 more structured discussions.

This structure can be more efficient when the CAB goals are to make concrete decisions on items that need a yes or no answer or to approve materials that are already created.

Benefits:

- Clearer delineation of roles and decisions
- May require less CHC staff time

Drawbacks:

- Less organic, in-depth discussions
- Requires additional effort from members who serve as chairs or co-chairs

Informal Structure- A CAB that functions with more loosely defined meeting rules and may not have a single, designated leader.

This structure is best when the CAB goals are to make broad decisions, give feedback on projects, and collaborate on materials development.

Benefits:

- More accessible for CHC's to set up
- More accessible for members to join as they do not need to learn or follow strict guidelines/rules during meetings
- More open discussions and flexibility in meetings

Drawbacks:

- Will require more effort of a staff coordinator to act as organizer and/or facilitator

Chair and/or Co-chair

- CABs often elect, nominate, or rotate a chair or co-chairs who serve as the CAB leader and work closely with a staff coordinator to achieve CAB progress, run meetings smoothly, and engage members.
- Note: CHC/Comm member co-chairs

Benefits:

- Variety of ways to implement this structure
- Structure is often used for other types of boards and may be familiar to members
- A CAB member serving as chair centers the community voices and may increase community trust

Drawbacks:

- Requires some members to dedicate more time/effort to CAB

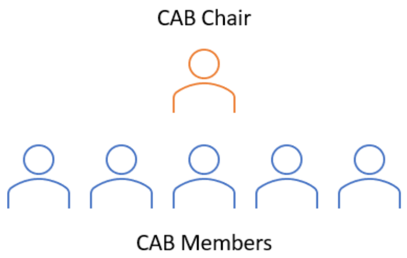

Example:

Chair = CHC Staff facilitator

- Prepares meeting agendas
- Prepares and sends communications

Co-Chair = CAB member

- Participates in agenda setting
- Helps to move meetings along and keep conversations focused
- “Taps in” other CAB members to engage

CHC-Led CAB

- A CAB without a community member leader will rely more heavily on staff to facilitate meetings, create agendas, and engage with other members. The CHC may choose to rotate facilitating duties between staff for each meeting or ask one person to permanently serve as the facilitator.
- A CAB member and CHC staff member may serve as co-chairs

Benefits:

- Does not add time/effort to CAB members

Drawbacks:

- Increased CHC staff time/effort required
- Increased role of staff may lead to a decreased community voice

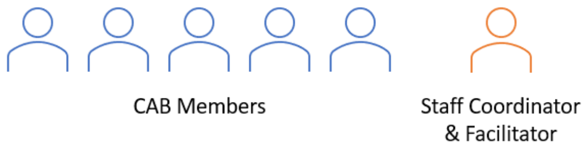

Example:

RADx CABs, focused on increasing COVID-19 testing, were run using this model. A staff member, or sometimes two staff members, led the board that was made up of community members. The staff members handled the administrative work of scheduling and notetaking, along with setting the agenda and facilitating meetings.

Importantly, these staff also served as a link between the CAB and health center leadership. They were able to take ideas from the CAB, discuss them with leadership, and then update the CAB members on the progress.

Steering Committee

- Steering committees are typically involved in setting the path forward for the work of an organization or community board. They identify the priorities of the group and the projects they will dedicate time and resources to.
- A Steering Committee could also be used to lead a large or complex CAB
- Some steering committees serve as the main leadership of the group, with subcommittees or working groups focused on more specific projects that report back to the steering committee.

Benefits:

- Many CHCs utilize steering committees within their work, such as in quality improvement or as part of the executive board, making this a natural model to adopt
- Community member led, promotes trust
- Allows members to gravitate toward their interests and passions to foster engagement
- Splits the time and effort of leadership across multiple people

Drawbacks:

- More difficult to implement, particularly with a smaller CAB
- Requires additional meetings, time, and money

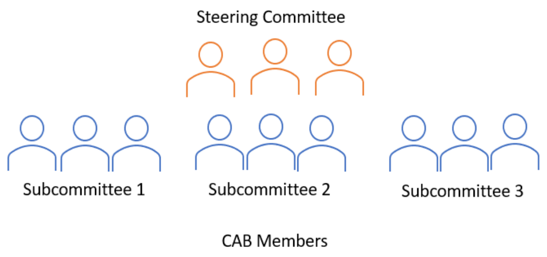

Example:

One CHC described how their team functions through using a steering committee and subcommittees. The steering committee identifies the priority projects to focus on and then creates a subcommittee that is focused on those project goals. These subcommittees are disbanded when the project is completed or have other staff rotate in for longer term projects. The CHC explained that this structure helps to keep people from burning out and is helpful for focusing on specific improvement goals.

Choosing a Chair

It is often difficult to determine how best to select chair(s) to lead the CAB. Be sure to establish the responsibilities and time commitment of the chair prior to beginning the selection process.

Voting	First, survey members to see if they are interested in serving as the chair. Create a list of interested members and ask all members to vote on the chair. Members can vote with a formal process during a meeting or via online poll.
Nomination	CABs may choose to ask members to nominate CAB members to serve as the CAB chair. Members can vote on nominees using a formal vote during a meeting, an online poll, or by identifying who is interested and asking the members to discuss and approve the appointment by consensus.
Rotating chairs	It may work best for your CAB to rotate between members who serve as the chair to prevent burnout and create leadership opportunities for more members. This rotation can be done several ways, including based on time or project goals. For example, chairs can be appointed at the beginning of a new project and rotated when major milestones are achieved. Alternatively, chairs may serve for a set period of time such as 1 year.

Key Takeaways

1. CABs should outline agreed upon communication, decision-making, prioritization, and conflict resolution strategies from the start.
2. The level of formality within a CAB structure should align with its purpose and goals.
3. Consider the pros and cons of each type of leadership structure and how they fit into existing CHC structures.

CAB Member Recruitment

Identifying and recruiting potential CAB members is an important step in CAB startup and is key to maintaining an active and vibrant board. This chapter will walk through how to identify and recruit members, get member agreements to participate, and orienting new members to the board.

Community members are busy with lots of competing priorities and time commitments. Communicating the ***value of being a CAB member*** is an important part of the recruitment process. This could include testimonials of personal experiences or stories of community impact as described below.

> Honestly, I think we built friendships.
>
> There are at least two people I talk every other week now. Even if it's questions about what I do and questions about what they do – we exchange ideas.
>
> This was only possible because we were a part of the CAB. If it wasn't for them, I don't know if we would cross paths.

> It was my first time being in advisory anything so just, learning myself to have more confidence in myself and in the confidence of my team and the people that I work with.
>
> I didn't quite understand why I was there at first. But there was a reason, and they had faith in me that I needed to be there, and I was the right person to be in that group.

> I probably would not have been brave enough to even go and contact the school department if it wasn't for some of my other CAB members. We gave each other the strength so we could get the results we needed at the end.

Example: Share Stories to Demonstrate Community Impact during Recruitment

The Issue: Several communities in the Greater Boston Area had low COVID-19 testing rates, despite high COVID-19 infection rates.

The Project: Community health centers created Community Advisory Groups through a grant, RADx, that focused on access to COVID-19 testing. The health center had a resource to use - a community health van.

The CAB 's Impact: The CAB members were instrumental in guiding the van's activities.

1. *Advised on how to best utilize the van as the COVID-19 pandemic progressed:* first for COVID-19 testing and then vaccination.

2. *Identified where the van should visit:* in areas where homeless people lived, at faith-based organizations, and at food distribution sites.

3. *Who should staff the van:* CAB members heard from community residents that other testing sites were not providing appropriate advice on isolating, protecting family members, and what to expect for COVID-19 test follow up. To fill this gap, a doctor or nurse was always present and was often accompanied by a CHC staff member who lived in the community.

4. What resources should they provide: they included comprehensive COVID-19 information and guidance depending on the results of the COVID-19 test. Patients left knowing when and how they would receive their results.

Identifying Members

CABs are generally between 6-15 members. The size of the CAB should align with the purpose. There are several different strategies that CHCs can use to identify members. The Community Toolbox recommends that after creating a list of potential candidates, CAB members and staff members take a step to review the list for completeness of both established and emerging community leaders.[1]

Smaller CABs	Larger CABs
• Best for CABs focused on a single issue<br>• Easier to manage discussions and meeting logistics<br>• Low attendance may lead to very small or cancelled meetings	• Best for CABs with a broader scope<br>• Allows for a CAB with a larger variety of lived experiences and profession fields<br>• Easier to maintain active CAB meetings even with lower attendance

Strategies to Identify Members	
Selection Committee	A CHC can form a selection committee that includes staff from various positions including CHWs, nurses, and providers to nominate patients and community members that would be effective CAB members.
Identifying members by topic area	CHCs may want to recruit members based on a specific topic. Topic areas might include: cancer prevention, immigrant health, housing, physical activity, or education. This approach can be helpful when asking current CAB members to nominate new members. Focusing on specific fields, populations, or settings may lead to more nominations rather than asking broadly.
Contacting CBO leaders	CHCs may ask CBO leaders to nominate community members
Potential Member Matrix [2]	CHC staff or CAB members can use this tool to compare potential new members based on their affiliations and strengths. (see Appendix B)

Recruiting Members

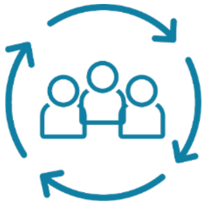

The CHC team and any existing CAB members should develop a clear message communicating the CAB's purpose prior to recruitment. Communicating a clear purpose to potential members can help to recruit the right members and set the board up for success. Potential members should understand what their role on the CAB is expected to be and why they are being asked to join. Once the CAB is established, it will be helpful to develop a mission and vision statement to communicate to new members who are recruited to fill vacancies. See Appendix C for guidance.

Recruitment materials should include:

1. CAB purpose statement: include why the group exists and what it is trying to achieve. This might include specific health issues, projects, or populations that the CAB is focused on.
2. Time commitment: include meeting frequency, time, location, and length of membership
3. Expectations of the potential CAB member: include what they will be expected to discuss during meetings, community events they will attend, engagement between meetings
4. Incentives or compensation (See budgeting section for more details)

Recruitment materials (e.g., one-pager, letter, brochure) may be distributed via email, physical mail, or in-person events. Some CHCs may choose to use phone calls or text messages. See Appendix D for examples of CAB recruitment materials.

Some CABs may choose to include an application or interview process for potential members. An application or interview can help to ensure potential members fit the characteristics the CAB is looking for, however they will require a larger time investment for members and CHC staff

Characteristics of Effective CAB Members

When you've identified potential new members, consider discussing the following question with them:

- *What level of time do you have to commit to the CAB?*
- *What topics are you particularly excited about working on?* [3]
- *What do you see as the priorities for the community?* [3]
- *What makes you feel connected to the community?*
- *What helps you to feel comfortable sharing your feedback and opinions in a group?*

Lastly, make sure that your recruitment team internally discusses whether the person is reflective of the patient population, demographically and experientially.

Member Agreements

It is important to provide a clear written invitation from a point of contact at the CHC or on the CAB. This person should serve as a resource to answer any questions about the CAB.[2]

As is the case with many components of this toolkit, the formality of the agreement should align with the goals and purpose of the CAB. Some CABs choose to draft Letters of Agreement, Memorandums of Understanding, or Charters. See an example of components to include in the member agreement in the figure to the right and examples of member agreements in Appendix E.

Member Onboarding

When recruiting members to an existing CAB to fill vacancies, CABs should consider pairing a new member with a more experienced member as a peer mentor. This model has been used successfully on CHC Executive Boards. These relationships can help new members understand the purpose of the CAB and their individual role. The experienced member can assist the new member in learning about current projects and ground rules as well as assist in building their confidence participating. This mentoring can foster trusting relationships and effective CAB impact.

1. Regular and ongoing meeting attendance
2. Ability to commit to membership for the length of the project
3. Provide input above a variety of issues related to the project
4. Open to including goals that may be beyond your specific interest (yet still relevant to the topic) that may be of interest to the group
5. Providing one-on-one consultations on areas of expertise

Key Takeaways

1. Clearly communicate with potential CAB members about the value to membership, the roles and expectations of members, and the purpose of the CAB.
2. Review the variety of ways to identify potential CAB members and select those that best fit your community and the CAB's needs.
3. When a potential CAB member agrees to participate, create an agreement with them that fits the formality and style of the CAB.

Meeting Logistics

Carefully considering the logistics of CAB meetings will help them run smoothly, ensure productivity, and respect the time and efforts of members. This chapter includes practical tips for scheduling, hosting, and following up on meetings.

We recommend identifying a staff coordinator to support the planning and logistics of CAB meetings. The coordinator can be someone in a leadership position at the health center or be built into staff roles such as QI managers or community health workers. Having one point of contact will help ensure consistency and clear expectations.

- Based on interviews with former CAB coordinators, the estimated time to plan meetings and outreach to members between meetings can range from 1.5 and 5 hours.
- The exact responsibilities of the coordinator will depend on the structure of the CAB and may shift over time.
- Typically, CHC staff coordinators will lead meeting scheduling and lead or assist CAB members in setting and sending meeting agendas, and creating any meeting materials such as slides.

The checklist below for planning a CAB meeting Use the checklist below to plan a CAB meeting. See Appendices F and G for a sample agenda and a meeting minutes template.

> While scheduling meetings at different dates/times is more flexible, it requires more communication between members and staff. Set meetings may help members prioritize attending but are inflexible to changing schedules.

Planning Checklist

One month before meeting:

- [ ] Poll members to pick a date and time that works best

> If you prefer a set time for each meeting, survey CAB members at the first meeting to set a date/time for the coming year

- [ ] Pick a convenient meeting location with options for parking, public transit, and/or bike parking
  - If virtual meeting: confirm member's internet access and comfort with videoconferencing platform
- [ ] Create meeting agenda
  - Be sure to include all action items from last meeting and how CHC has addressed them
  - Review agenda with CHC chair or co-chair

> If your CAB meets virtually, consider 1 in-person meeting per year for relationship building.

- [ ] Send meeting invites via email, calendar invite, or text. Include Zoom link or physical address.
- [ ] Order refreshments/food, preferably from local businesses

One week before meeting:

> It is important to understand CAB members preferences for communications and can be established when members first join

- [ ] Prepare meeting slides and handouts
- [ ] Send meeting reminders, including meeting agenda

Two weeks after meeting:

- [ ] Send meeting minutes
- [ ] Distribute participant payments and transit reimbursements

Key Takeaways

1. Well run and organized meetings will help ensure your CAB is effective and respected.
2. The checklist above is a starting point for planning.

Meeting Facilitation

Facilitating meetings with a diverse group of CAB members is an important role. Successfully leading a CAB meeting can enhance member's satisfaction with and the effectiveness of the CAB. Below, you will find a set of best practices that have been collected from existing resources and input from community and health center partners.

Best Practices
**Before the Meeting:**<br>1. Create a meeting agenda and share it with all CAB members (see Appendix F)<br>• Balance meeting objectives and what it is realistic to cover given the time<br>• Build in time at the beginning of the meeting for members to socialize and introduce new members<br>• Include time for group discussion and questions during the meeting<br>• Review meeting minutes from last meeting and address any items that required follow-up<br>2. Consider reaching out to quieter members ahead of time if you'd like their input on a specific topic<br>3. Designate a note-taker and the preferred template or form for taking notes (See Appendix G)
**During the Meeting:**<br>1. Start the meeting on time and remind attendees of the meeting end time<br>2. During initial meetings, establish ground rules for engagement to provide shared expectations on how the group will work together and serve as a reminder of the values the group intends to uphold. Revisit annually.<br>3. If new members are attending, consider an ice-breaker for the group<br>4. Follow the agenda, but be flexible to allow for discussion and questions<br>5. Observe quieter members and consider how best to engage them<br>6. Pause to ask members if everyone agrees with the points that have been made and what questions members want to ask<br>7. Ask open-ended discussion questions and focus on one topic at a time<br>8. Allow time for members to share updates and ask members for advice<br>9. Clearly note which items require follow-up at next meeting
**After the Meeting:**<br>1. Finalize and send meeting minutes to all members with clear action items including deadlines and roles. The CAB should have an agreed upon schedule they follow for sending meeting minutes.

Key Takeaways

1. Be prepared for a CAB meeting with a clear idea of the agenda, engage members in a way that works best for your community, and be sure to follow up with CAB members with the meeting minutes.

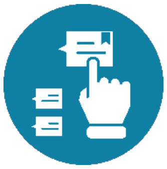

Prioritization

Why use a prioritization strategy?

CHCs are faced with many health issues that are important to patients and the broader community. Choosing where to focus limited resources is critical for making meaningful impact. CABs should balance factors such as the cost of addressing the health issue with the urgency to take action. What you are prioritizing may change over time but can include *health topics of focus, goals* for the year*, priority populations,* and *solutions* for health issues.

Using a structured, clear strategy to prioritize can be both efficient and facilitate engagement. In this chapter, you will find different approaches to prioritization including specific strategies, materials, and ways to harness CHC resources to assist with these efforts.

Before Prioritization

Data gathering

Before beginning the prioritization process, CABS should work with the CHC to gather data on the community and patients.

> Hospitals are required to complete community health needs assessments every 3 years. These are great local sources of data.

- Potential data sources:
    - Community Health Needs Assessments
    - Data from Electronic Health Records
    - QI metrics
    - City/county level health from the population surveys
- Reviewing this data together will help the CAB approach prioritization with a shared understanding of community health outcomes.
- If there is a gap in the data, CABs may collect their own data such as surveying patients or interviewing community members.
- QI team members could present data on key health outcomes, either in a meeting or through a brief 1-page overview document.
- Data gathering is an opportunity to identify disparities and discuss solutions to drive equity.

Select Prioritization Criteria

Harnessing QI Data

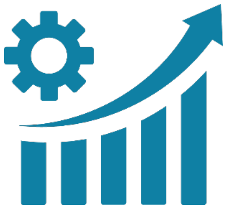

- CHCs with a quality improvement team can harness QI metrics as a data source to use before prioritization.
- CABs may request QI teams to identify QI metrics that need improvement through a one-page report or a brief meeting presentation. CABs can then apply a prioritization tool to decide between which metrics to address.
- QI metrics and population health data are an opportunity to identify disparities and discuss solutions to drive equity.
- QI metrics can be applied as prioritization criteria throughout a prioritization process.
- Examples of metrics to use as criteria: the magnitude of gap between current status and goal for the QI metric, severity of disease, cost of treating patients. See Appendix H.

CAB members should agree on which criteria will help inform decisions before beginning the prioritization process. The criteria your CAB selects as the most important may change over time or with each project. For example, one approach might be for CABs to review the list of potential criteria and select two that are most important on an annual basis.

Potential Criteria	
Cost	• How expensive will be to address this need? Who bears the cost burden?
Available solutions	• Are there evidence-based practices or policies to address this need?
Resources	• Do you have the staff, time, money, & equipment to address this need?
Urgency	• What is the severity of impact of the need?
Size	• What is the burden of disease in population? Among priority groups?

Prioritization Strategies

After agreeing on criteria, your CAB should agree on which prioritization strategy to use. Review the considerations for several commonly used prioritization methods below. The NACCHO Guide to Prioritization Techniques [5] and Do Tank [6] provide step-by-step guidance and tools to make prioritization easy and fun!

Prioritization Strategy	Purpose, Process, Benefits & Drawbacks
**Multi-voting**	**Purpose:** Identify the issues with greatest support<br>**Process:** Members narrow down a list of health issues through several rounds of voting<br>**Benefits:** Voting in anonymous and equal vote is given to each participant<br>**Drawbacks:** No discussion to share experiences and understand diverse perspectives
**Nominal Group**	**Purpose:** Use group discussion to generate ideas and prioritize issues<br>**Process:** A moderator leads brainstorming session and ranking<br>**Benefits:** Includes opportunity for sharing perspectives and equal votes for all participants<br>**Drawbacks:** Difficult to facilitate
**Strategy Grid**	**Purpose:** Identify greatest opportunity for impact<br>**Process:** Members select two criteria (cost/impact, community support/feasibility, etc.) and create a 2x2 table with high and low where they categorize each topic<br>**Benefits:** Collaborative process<br>**Drawbacks:** Limits considerations to two criteria and not all voices will be weighed equally

Key Takeaways

1. Prioritization strategies can help your CAB choose which areas of health, populations, or programs to focus on.
2. Your CAB will need to review some data and select prioritization criteria before beginning the process.
3. Review the benefits and drawbacks of each prioritization strategy to select one that aligns your CAB the goals.

Cost and Sustainability

How much does it cost? This is a common question when it comes to starting up a new initiative within health centers and important for ensuring long term impact. This chapter provides an overview of the costs you can anticipate for planning and implementing a CAB.

Budgeting for Staff Time

These costs will largely fall under staff time allocated for supporting CAB activities, so you may want to consider your staff leadership structure (see pages 5-7) and support staff roles with cost in mind. These staff costs may not be additional line items in a budget, but it is important to account for time staff may need to shift to support CAB success. Examples of activities involving staff time include:

Planning costs

- CAB member outreach and recruitment
- Planning CAB structure and logistics

Implementation costs

- Planning meetings
- Facilitating meetings
- Notetaking, scheduling, and other meeting support
- Other staff participating in meetings
- Outreach and communication between meetings

Additional Items for your CAB Budget

Other costs besides staff time might include CAB member incentives (e.g., stipends) and transportation reimbursement, food and drink, meeting space fees, and printing materials for in-person meetings.

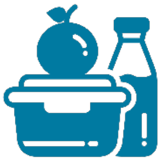

Food and drink – There is value in sharing a meal while gathering for in-person CAB meetings. Food could include coffee and snacks for morning or afternoon meetings or a full lunch if hosting mid-day. We recommend catering purchased from a small local business.

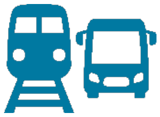

Transportation – make sure to cover any parking costs or transit far for in person meetings. If people are travelling from afar, offer mileage reimbursement to compensate them for the drive.

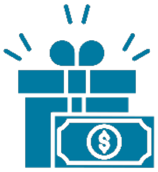

CAB member incentives – Members should be compensated for the time and effort they contribute to the CAB. See more tips below!

Case Example – RADx MA

The Rapid Acceleration of Diagnostics for Underserved Populations – Massachusetts (RADx-MA) project was a research grant that supported CHC-community partnerships in nine hotspot communities to promote equitable access to COVID-19 testing. This included development of CHC-led CABs. Below are examples of cost estimates for planning and implementing these groups. The pandemic limited meetings to virtual Zoom calls, so the primary costs are staff time. The

table details the types of costs and staffing structures for each of these four CABs. The annual estimated cost for a CAB averaged about $8,000. Given the urgency of the pandemic, these CABs chose to meet monthly. However, with a quarterly meeting structure CABs would likely cost under $5,000 annually.[1]

	Example 1	Example 2	Example 3	Example 4
**Estimated annual cost (monthly mtgs)**	$6,680	$7,730	$8,250	$10,510
Cost by activity				
**Planning**	$1,280	$450	$1,080	$1,400
**Implementation**	$5,400	$7,280	$7,170	$9,110
Cost by resource type				
**Paid Labor**	$6,680	$6,530	$8,250	$8,850
**Member incentives**	-	$1,200	-	$1,660
**Staff leading activities**	Facilitator - Clinical Operation Director	Facilitator - Clinical Director<br>Facilitator - Operations Manager	Facilitator - VP Research & Population Health	Facilitator - COO<br>Coordinator - CHW

Member Compensation

We strongly encourage budgeting resources to provide incentives to show you value the time, experiences, and expertise CAB members contribute. Examples of member compensation include gift cards ($25-$50 per meeting), annual stipends ($350-500) per person or organization issued, and small gifts or celebrations to show appreciation. By making this clear in your recruitment efforts, you may also improve your chances of acceptance and more diverse representation.

People making over $600 per year are required to report their income to the IRS, which may have implications for administrative burden at your health center or willingness to be paid among some CAB members. We recommend tailoring incentive distribution method to acknowledge privacy and access concerns, particularly among members who are immigrants or those with unstable housing. Offer members the option to receive compensation by email, at their home address, or have it sent to a trusted community organization, so they don't have to disclose personal information.

[1] In this case example, costs were estimated using interviews to identify staff involved, time spent on specific tasks, and non-labor costs. We used salary estimates specific to the state of MA provided in the National Association of Community Health Centers' Health Center Salary & Benefits Report 2021-2022 and assumed a 28% fringe rate.

Funding Sources

Determining funding sources is critical for sustaining CAB efforts.

In some cases, it may be feasible to leverage grant funds to offset associated costs. You might consider creating expectations for collaborators that working with your CHC will involve CAB input, including budgets that include CAB costs. A budget template is included in Appendix and can be downloaded as an excel file.

Other potential funding sources to explore include quality-based care funds under Accountable Care Organizations and value-based care contracts. These approaches have been used to support community health workers and could be an innovative mechanism for ensuring financial support.

Finally, CHC executive boards may add resources to an operational budget to support CAB activities. This could involve including CAB facilitation into specific job descriptions to sustain these roles regardless of any organizational turnover or specific line items for CAB implementation.

Key Takeaways

1. The cost of planning and implementing at CAB primarily consists of staff time. Other costs to budget for include CAB member incentives, transportation reimbursement, food and drink, and printing materials.
2. Among health centers that have implemented successful CABs in the past, costs averaged about $8000 per year when groups met monthly. Costs would be around $5000 for CABs that meet quarterly.

Evaluation

Collecting evaluation data on engagement of your CAB members may be valuable for internal quality improvement efforts and demonstrating to funders the value of engagement. In this chapter, you will learn about several suggested strategies for collecting data on your CAB that are both feasible and useful.

Surveys

Surveys of CAB members can help to quantify the level of engagement. The Research Engagement Survey Tool is an example of a brief validated survey for measuring the frequency and quality of partner engagement. While this tool was originally designed for use with CABs operating within a research context, it has been used for understanding CHC-based CABs too. The survey could be used to assess engagement on a quarterly or annual basis to track progress over time with opportunities to discuss results and strategies for improvement as a group. Alternatively, the survey could be fielded at the beginning and end of a large project as data to demonstrate CAB impact or as new members cycle onto a CAB to ensure high quality sustained engagement over time.

Examples of the brief survey adapted for a CHC-based CAB, a long form survey, and scoring instructions can be found in Appendix I. More information is also available here: https://wp.nyu.edu/collegeofglobalpublichealth-goodman_mle_lab/rest

Interviews

One-on-one interviews with CAB members can help to gather more in-depth feedback on engagement. These could be done in person or remotely and are often best conducted by someone who does not lead the CAB meetings to encourage candid responses. Your CHC may choose to conduct interviews after the first year or annually to identify areas for improvement and success stories.

Ideas for interviews questions include:

- Why did you decide to join the community advisory board?
- What do you see as the greatest strengths of the community advisory board?
- What are the greatest challenges the community advisory board has faced this year?
- Thinking about the community advisory board meetings this year, were there aspects of the meeting that worked well? Needed improvement?
- If you could speak to a CHC leader who is about to initiate their first community advisory board, what advice would you give them?

Key Takeaways

1. Surveys can help quantify the level and quality of CAB engagement. We encourage using a validated tool like REST to ensure accurate assessment.
2. Interviews help you understand the experiences of CAB members in greater depth, which can be useful for identifying successes and areas for improvement.

Appendices

A. Training on Conflict Resolution

Community Toolbox

This resource includes an overview of what conflict Resolution is as well as why, how, and when it should be done. Tools include questions to determine the interests of multiple parties (screenshot below).

https://ctb.ku.edu/en/table-of-contents/implement/provide-information-enhance-skills/conflict-resolution/main

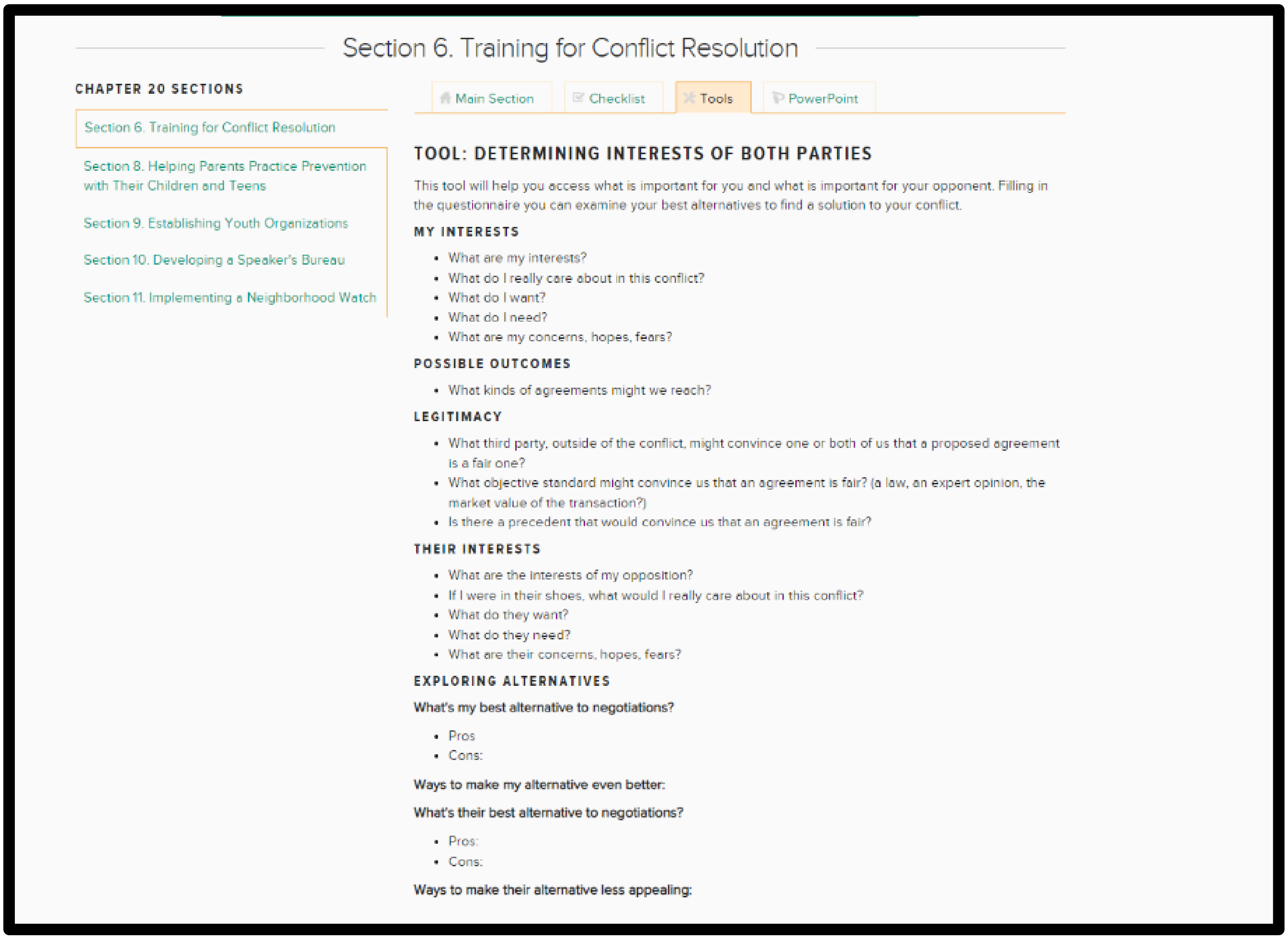

Section 6. Training for Conflict Resolution

CHAPTER 20 SECTIONS

Section 6. Training for Conflict Resolution

Section 8. Helping Parents Practice Prevention with Their Children and Teens

Section 9. Establishing Youth Organizations

Section 10. Developing a Speaker's Bureau

Section 11. Implementing a Neighborhood Watch

Main Section | Checklist | Tools | PowerPoint

TOOL: DETERMINING INTERESTS OF BOTH PARTIES

This tool will help you access what is important for you and what is important for your opponent. Filling in the questionnaire you can examine your best alternatives to find a solution to your conflict.

MY INTERESTS

- What are my interests?
- What do I really care about in this conflict?
- What do I want?
- What do I need?
- What are my concerns, hopes, fears?

POSSIBLE OUTCOMES

- What kinds of agreements might we reach?

LEGITIMACY

- What third party, outside of the conflict, might convince one or both of us that a proposed agreement is a fair one?
- What objective standard might convince us that an agreement is fair? (a law, an expert opinion, the market value of the transaction?)
- Is there a precedent that would convince us that an agreement is fair?

THEIR INTERESTS

- What are the interests of my opposition?
- If I were in their shoes, what would I really care about in this conflict?
- What do they want?
- What do they need?
- What are their concerns, hopes, fears?

EXPLORING ALTERNATIVES

What's my best alternative to negotiations?

- Pros
- Cons:

Ways to make my alternative even better:

What's their best alternative to negotiations?

- Pros:
- Cons:

Ways to make their alternative less appealing:

B. Creating a Resourceful and Diverse Community Advisory Board

University of Arizona Center for Indigenous Environmental Health Research

The purpose of this worksheet is to create a list of potential CAB members who have different backgrounds and roles in the community.

› Sectors and individuals will vary according to the goals of the project.

› Individuals may be selected based on some of following characteristics:

1) place of employment
2) place of residence
3) reputation or leadership in the community
4) recreational or social activities in the community
5) any potential conflict of interest.

› A conflict of interest might include a community leader or business that would benefit financially or politically from advocating for promoting a project. The project team should consider if a CAB member's benefits outweigh the community benefits.

THE UNIVERSITY OF ARIZONA
MEL & ENID ZUCKERMAN COLLEGE OF PUBLIC HEALTH
Center for Indigenous Environmental Health Research

Worksheet 1.2: Creating a Resourceful and Diverse Community Advisory Board

The purpose of this worksheet is to create a list of potential CAB members who have different backgrounds and roles in the community. A CAB should include representation from different community sectors that may play an important role in the success of a project. Use the table below to identify potential members of the CAB by each sector. Be prepared to share your ideas with the group.

Sectors	Perspectives Needed for the Project from Each Sector	Individuals Who May Represent Each Sector	Recruitment Strategies for Each Sector	Potential Conflicts of Interest or Barriers to Recruitment for Each Sector or Individual
Youth (individual 18 years or younger)				
Parent				
Elder				
Business				
Media				
School				

C. **Developing Vision and Mission Statements**

Community Toolbox

This resource helps users learn how to develop effective vision and mission statements to effectively communicate the work of their organization or effort. It includes an overview of what these statements are as well as why and how to create them. Tools include examples and the worksheets below.

https://ctb.ku.edu/en/table-of-contents/structure/strategic-planning/vision-mission-statements/main

Considerations for developing your Vision Statement:

- A vision statement is a statement about ideal conditions or how things would look if the issue important to you were completely, perfectly addressed.
- Common characteristics of vision statements:
    - Understood and shared by members of the community
    - Broad enough to include a variety of local perspectives
    - Inspiring and uplifting to everyone involved in your effort
    - Easy to communicate- for example, they are generally short enough to fit on a T-shirt
- Examples:
    - Caring communities
    - Safe streets, safe neighborhoods
    - Health for All

Instructions:

1. Identify one person to take notes while the group brainstorms ideas and one person to document the decision reached through consensus
2. Ask the following questions, record key points, and discuss common themes: (30 minutes)
    a. *Essential why*: What is the dream or ideal that you and your community seek?
    b. *Essential what*: What would have to change for this dream to come true?
3. Come to consensus about what the vision statement should be by considering the following: (10 minutes)
    a. Will it draw people to the common work?
    b. Does it give hope for a better future?
    c. Will it inspire community members through positive, effective action?
    d. Does it provide a basis for developing the other aspects of your action planning process?

Considerations for developing your Mission Statement:

- A mission statement describes *what* the group is going to do and *why* it is going to do that.
- Guiding principles for mission statements include:
    - *Concise*. Mission statements generally get their point across in one sentence.
    - *Outcome-oriented.* Explain the fundamental outcomes your organization is working to achieve.
    - *Inclusive*. Make broad statements about your groups' key goals but are not limiting to specific strategies or sectors of the community.
- Examples:
    - Promoting child health and development through a comprehensive family and community initiative.
    - To develop a safe and healthy neighborhood through collaborative planning, community action, and policy advocacy.
    - Promoting community health and development by connecting people, ideas and resources.

Instructions:

1. Identify one person to take notes while the group brainstorms ideas and one person to document the decision reached through consensus.
2. Carry forward ideas generated in developing your vision statement. Gather the ideas generated that described the "*essential why"* or the dream/ideal you seek and the *"essential what"* or what would have to happen for the dream to come true.
3. As a group select the statements that have particular relevance for the vision statement identified and brainstorm potential mission statements (e.g. Our mission is to _______________ (essential why) through (or by)_____________________ (essential what). (30 minutes)
4. Come to consensus by considering the following: (10 minutes)
   a. Does it describe the *what* your group will do and *why* it will do it?
   b. Is it concise (one sentence)?
   c. Is it outcome oriented?
   d. Is it inclusive of the goals and people who may become involved in the work?

Record the agreed upon vision and mission statements below:

The Vision of our initiative is:

The Mission of our initiative is (*the essential why*):

through (or by) (*the essential what*):

D. Recruitment Materials

The following example of a recruitment email provides language to draw from when you conduct outreach to new members.

> Dear {insert name],
>
> I hope all is well with you! {Insert warm sentence connect referencing existing connections].
>
> I'm writing to extend an invitation for you to join the Community Advisory Board for the [insert health center with link]. Our center's mission is to [insert]. [Insert 2-3 sentences about patient population and community context}.
>
> We are currently looking to expand the perspectives on our Community Advisory Board that helps to guide how our team conducts our research and engages with partners in implementation of prevention efforts at our Center. The group currently meets on a quarterly basis and comprises of state and local health agency leaders, stakeholders from the Boston Public Schools and Department of Elementary and Secondary Education, community-facing hospital representatives, and the Massachusetts Public Health Association leadership [adapt to types of roles currently on CAB]. At our last meeting, the group expressed interest in bringing in people from the childcare, community development, transportation, and small business sectors [adapt to align with process of selecting members]. We'd love to invite you to join the Community Advisory Board to share your experiences addressing issues of health equity within community development spaces across the state [adapt to experiences of new member]. I hope you are interested and would love to set up a time for us to discuss more – please let me know if there are some times that might work best for you in the coming weeks.
>
> Best,
> [insert CAB leader name]

E. Member agreement materials

The following example Memorandum of Understanding and Charter provides language to draw from when developing agreements for your CAB.

Example 1: Memorandum of Understanding

Regarding the Community Advisory Board at the [Insert Health Center Name]

This Memorandum of Understanding ("MOU"or "agreement") effective on **[insert date]** is between [**insert Health Center name]** located at **[insert Health Center address, city, zip code]** and [**Name of CAB Member Organization or Person** at address, city, zip code} ("CAB Member"). This MOU describes the understanding between the parties regarding the HPRC Community Advisory Board (CAB).

The mission of the **[insert health center]** is to work with communities, community agencies, state and local government, and other partners to develop, implement, and evaluate methodologies and interventions to improve nutrition and physical activity and reduce overweight and chronic disease risk among children, youth, and their families, and to reduce and eliminate disparities in these outcomes. The Community Advisory Board will support this mission as a shared goal.

Accordingly, the parties agree as follows:

A. **Terms-**
   a. Initial appointments are for two years, with the option to continue participation for the full five year project period.
   b. Full meetings of the board will occur quarterly each year.
   c. CAB members are expected to attend 50% of meetings each year. If participation falls below this level, membership will be revisited.
   d. Between meetings, board members will have opportunities to get involved with specific projects throughout the year.

B. **Shared Leadership Roles-**
   a. CAB members will collaborate with the center, by:
      i. participating in quarterly meetings,
      ii. selecting two members to co-chair the board with staff, this includes helping to identify topics, jointly setting agendas, and co-facilitating meetings as well as championing the Center locally and nationally (e.g., disseminating information across networks, participating on national community committee calls)
      iii. participating in broad strategy development and implementation. Such strategies include who to work with, how to get started, what procedures to use, and where to site projects.
      iv. keeping the Center informed and in tune with community perceptions of our work and providing guidance through discussion and networking.
      v. generating and assisting with development of ideas for new projects.
      vi. assisting with developing community-appropriate dissemination strategies.
      vii. assisting with assessing community needs and strengths related to physical activity and nutrition among children, youth and families.
      viii. educating and increasing awareness about the Center's ongoing work and activities.

   b. The Center will:
      i. engage the CAB in creating quarterly meeting agendas
      ii. share briefings/materials to be reviewed at least one week before scheduled CAB meeting
      iii. schedule CAB meetings at least 2 months in advance
      iv. document and share meeting notes with CAB members
      v. provide a space for quarterly CAB meetings, including options for virtual connection, and reimburse CAB member parking/transportation costs
      vi. co-facilitate the quarterly CAB meetings
      vii. provide the CAB with updates regarding the Core Research Project
      viii. seek input and ideas from the CAB for the implementation of the Core Research Project, dissemination of research findings, tools and resources, the annual Community Partners Meeting and new projects
      ix. provide the CAB with Leaders in Health application materials for dissemination amongst member networks inform the CAB of opportunities for involvement related to additional projects

C. **Opportunities for Collaboration-** CAB members have the option to participate the the following activities:
   a. contribute to the Center's annual Community Partners Meeting. CAB members will have the opportunity to advise on the content of the meeting based on the interests and needs of various community members.
   b. engage in strategic planning for the Center research and translation agenda and translation plan. This process will be guided by input and ideas generated at the Community Partners Meeting.
   c. assist in refining the structure and content of the Leaders in Health Program, including incorporate concepts of cost-effectiveness analysis into the program.
   d. participate in research dissemination events, including providing input on event format
   e. advise on development of case studies to engage current and future leaders in public health on concepts of cost-effectiveness analysis for obesity prevention
   f. explore ideas for expanding the CAB to ensure engagement and diverse representation annually
   g. identify at least three areas for reciprocity (e.g., ways Center can support the work of CAB members) each year

D. **Outcomes-** The CAB will help with Center efforts to:
   a. Improve our understanding of organizational decision making related to implementing initiatives to improve physical activity, nutrition and obesity prevention.
   b. Disseminate and advance knowledge of the cost effectiveness of a range of strategies to reduce obesity prevalence.
   c. Become familiar with the network of current providers in the state.
   d. Create a public voice on the issues.
   e. Translate research to practice and policy.
   f. Facilitate the widespread use of evidence-based programs and policies.

Example 2: CAB Charter

Community Advisory Board (CAB) Roles and Composition

The Center seeks to increase the pace of adoption of evidence-based programs and policies to promote health, prevent disease, and eliminate disparities. In alignment with community-identified needs and interests, the program strives to achieve this goal through a combination of implementation science, policy research, and community partnerships. The program aims to create mechanisms for high-quality engagement with community and policy stakeholders, working collaboratively with partners to identify opportunities for accelerating population-level outcomes while providing robust research and training opportunities in community translation and increasing the pipeline of well-trained individuals interested in this field. The program's Community Advisory Board plays a critical role in representing community perspectives to guide program activity.

Advisory Board Roles

The CEP Advisory Board:

1. Fosters communication and linkages between the Center and community partners and stakeholders.
2. Identifies gap areas and pressing research translation needs in the community.
3. Identifies opportunities for the Center and the Advisory Board to be involved in other projects.
4. Assists with the development of criteria and the selection of sites for CEP projects and grants.
5. Ensures adherence to core values of community-engaged research, including culturally competent approaches to working with the community.
6. Reviews and give input on Center priorities, focus areas, and resource allocation.

Composition, Leadership, and Terms of Service

The Advisory Board is a group of individuals representing community-based organizations, state and local departments of public health, community health centers, hospital community benefits departments, public policy and advocacy organizations, and other stakeholder entities as appropriate. Operating under the current structure, the Advisory Board reaches decisions by consensus. The Advisory Board leadership will be comprised of a single Chair whose main tasks are planning and co-facilitation of quarterly meetings and conference calls, supported by Center leadership and staff. The Center Advisory Board Chair also represents fellow Advisory Board members in other Center forums and activities upon invitation. The stipend is a $2,000 honorarium per year. The Advisory Board will meet on a quarterly basis for approximately 1.5-2 hours per meeting; additional meetings and conference calls may be scheduled as needed. Meetings will be held on the first Friday of each quarter between 11:00 a.m. and 1:00 p.m. at a space secured by the Center or virtually/remotely. Advisory Board members will receive a stipend of $300 per quarterly meeting attended, payable to the individual CAB member or to his/her organization*;* the Center will also reimburse for parking/mileage and will provide lunch when meeting in person. Meetings will be recorded for archival purposes and minutes of each meeting will be made available to members by e-mail within 30 days.

Expectations of the CAB

Advisory Board members will serve for a term of three years with the option to renew their membership for consecutive three-year term upon mutual agreement with the Center; terms will be staggered to ensure the stability of Advisory Board membership during transition periods. The Center will provide members with formal letters of notification upon the commencement of new terms, the renewal of terms, and the conclusion of terms. Members are expected to attend three out of four quarterly meetings per year, either in-person or remotely, and if unable to participate themselves, may designate a colleague to attend on their behalf with prior notification provided to the CAB Chair and Center staff. Members are expected to assist with some inter-meeting tasks, such as document review or taking part in relevant meetings/calls as needed. Members serve at the discretion of the Center and the Advisory Board Chair and may be asked to step down if frequent absences occur.

Expectations of the Center

The Center recognizes that the Advisory Board is a valuable group of individuals whose expertise is matched and indeed derived from their commitments to other endeavors. The Center will therefore make every effort to ensure that meeting time is used efficiently and respectfully, that the CAB is given the opportunity and encouraged to provide meaningful input, and that inter-meeting requests are kept to essential matters only that require CAB feedback or decision-making. The Center will also make every effort to align community-identified priorities, both raised by the CAB and identified elsewhere, with its own specific priorities. To ensure that CAB feedback is respected and duly considered, the Center will develop meeting agendas collaboratively with CAB leadership and will include in each meeting a review of "old business," updating the CAB on the status of previously suggested action steps. Lastly, the Center will provide meeting materials in advance (one month for minutes and one week for other items) so that members have sufficient time to review them prior to meetings.

F. Example Meeting Agenda

Time	Agenda Item	Facilitator
11:00 – 11:15	Welcome and Introduction<br>Approval of March Meeting Minutes	CAB Chair
11:15 – 11:45	Ambassador Program<br>• Overview of pilot plans<br>• Review job description<br>• Discussion: How can the Community Engagement Program best support Ambassadors and plan against unintended consequences (e.g., carrying burden of representing the community, addressing unfair practices)?	Center Assistant Director
11:45 – 12:00	New Funding Opportunity<br>• Social determinants of health interventions<br>• Community responsive projects<br>• Building on successes<br>• Partnership with new CAB organization	Center Director
12:00-12:15	Discussion: How can the Community Engagement Program improve incorporation of community members' lived experiences into our services?	CAB Chair
12:15 – 12:30	Program Updates<br>CAB Member Update	Center Assistant Director

G. Template for Meeting Minutes

Southern California CTSI Community Advisory Board Toolkit

https://sc-ctsi.org/uploads/resources/CommunityAdvisoryBoard_Toolkit.pdf

Appendix E: Note Taking Template

Date		Group	
Time Started		Session Number	
Time Ended		Facilitator	
Number of Participants		Co-Facilitator	

Question/Discussion [Each question should have a separate area for notes]

**Brief Summary/Key Points**	**Notable Quotes**
**Comments & Observations**	

Resource for Integrating Community Voices into a Research Study 29

H: Example of Applying QI to Strategy Grids

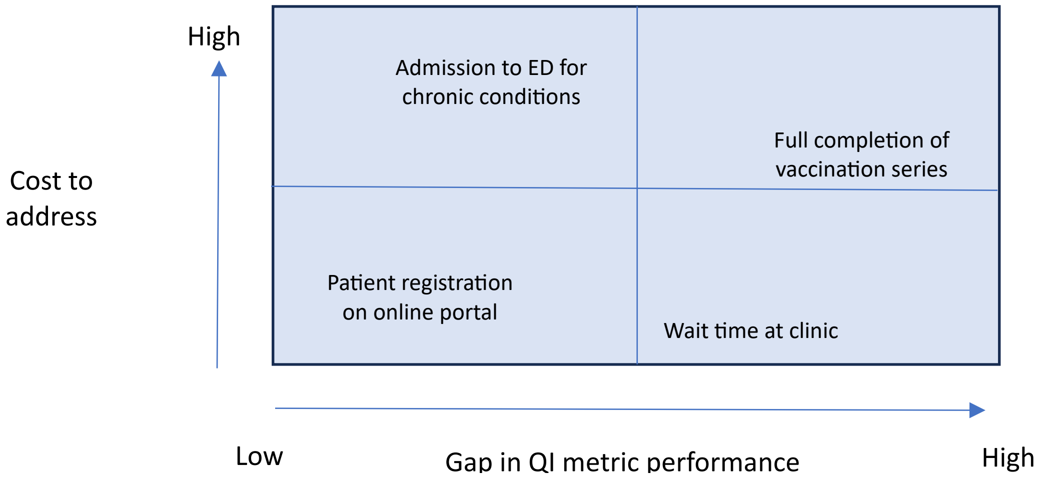

I: Research Engagement Survey Tool (REST)

CHC-based 9-item adaptation

Please think about your experience participating in the Community Advisory Board at your local health center. For the survey items below, answer the questions about your thoughts and experiences working on/participating in that project.

Please rate *how well* the partners leading the project do each of the following: (rate on a 5-point scale from 1-5: Poor, Fair, Good, Very Good, Excellent, NA)

1. The focus is on problems important to the community.
2. All partners assist in establishing roles and related responsibilities for the partnership.
3. Community-engaged activities are continued until the goals (as agreed upon by all partners) are achieved.
4. The partnership adds value to the work of all partners.
5. The team builds on strengths and resources within the community or patient population.
6. All partners' ideas are treated with openness and respect.
7. All partners agree on the timeline for making shared decisions about the project.
8. The partnership's processes support trust among all partners.
9. Mutual respect exists among all partners.

Now, please rate *how often* the partners leading the project do each of the following: (rate on a 5-point scale from 1-5: Never, Rarely, Sometimes, Often, Always, NA)

10. The focus is on problems important to the community.
11. All partners assist in establishing roles and related responsibilities for the partnership.
12. Community-engaged activities are continued until the goals (as agreed upon by all partners) are achieved.
13. The partnership adds value to the work of all partners.
14. The team builds on strengths and resources within the community or patient population.
15. All partners' ideas are treated with openness and respect.
16. All partners agree on the timeline for making shared decisions about the project.
17. The partnership's processes support trust among all partners.
18. Mutual respect exists among all partners.

Final Comprehensive Research Engagement Survey Tool (REST)

EP1. Focus on community perspectives and determinants of health

The focus is on problems important to the community.
All partners look at the data to determine the health problems the community thinks are important.
The effort incorporates factors (for example—housing, transportation, food access, education, employment) that influence health status.
The focus is on cultural factors that influence health behaviors.

EP2. Partner input is vital

All partners have the opportunity to share ideas, input, and leadership responsibilities and to share in the determination of the project structure.
Plans are developed and adjusted to meet the needs and concerns of the community or patient population.
All partners agree to take on specific tasks according to their comfort, ability, and expertise.
All partners assist in establishing roles and related responsibilities for the partnership.

EP3. Partnership sustainability to meet goals and objectives

All partners share updates, progress, strategies, and new ideas regularly.
A plan is in place for ongoing problem-solving.
All partners are involved in determining next steps.
Community-engaged activities are continued until the goals (as agreed upon by all partners) are achieved.
All partners continue community-engaged activities beyond an initial project, activity, or study.

EP4. Foster co-learning, capacity building, and co-benefit for all partners

All partners have a variety of opportunities to gain new skills or knowledge from their involvement.
All partners are encouraged to learn from each other.
The partnership adds value to the work of all partners.
All partners share resources to increase ability to address the problem of interest.

EP5. Build on strengths and resources within the community or patient population

The team builds on strengths and resources within the community or patient population.
The team works with existing community groups and organizations.
The team includes representation from the local community or patient population.

EP6. Facilitate collaborative, equitable partnerships

Fair processes have been established to manage conflict or disagreements.
All partners' ideas are treated with openness and respect.
All partners agree on the timeline for making shared decisions about the project.
All partners agree on ownership of data for publications and presentations.

EP7. Involve all partners in the dissemination process

All partners can use knowledge generated from the partnership.
All interested partners are involved in activities related to sharing results.
All partners have the opportunity to be coauthors when the work is published.

EP8. Build and maintain trust in the partnership

The partnership's processes support trust among all partners.
All partners are confident that they will receive credit for their contributions to the partnership.
Mutual respect exists among all partners.
All partners respect the population being served.
All partners understand the culture of the organizations and community(ies) involved in the partnership.

REST instructions

Notes on how measure is presented:
- The titles of the engagement principles (EP) are in bold text – these are not administered as part of REST
- The item(s) corresponding to each EP are presented below the corresponding EP title

Current measure prompt/intro:
Please think about your experience participating in: [list project description here] For the rest of this survey answer the questions about your thoughts and experiences working on/participating in that project.

Question Stems:
Quality: “Please rate how well the partners leading the research do each of the following.”
Quantity: “Please rate how often the partners leading the research do each of the following.”

Response Options:
Quality: Poor (1), Fair (2), Good (3), Very Good (4), Excellent (5), Not Applicable.
Quantity: Never (1), Rarely (2), Sometimes (3), Often (4), Always (5), Not Applicable.

REST scoring

Calculating Mean Scores: By EP and Overall
- Calculate EP specific scores as an average of non-missing items
- Then, average EP specific scores for an overall REST score
- This is done separately for the quality scale and the quantity scale; you will have a quality & quantity
- version of each EP and an overall score

Resources

CAB Member Recruitment

Creating and Maintaining Coalitions and Partnerships. 1. Creating and Maintaining Coalitions and Partnerships | Community Toolbox. Accessed September 5, 2023. https://ctb.ku.edu/en/creating-and-maintaining-coalitions-and-partnerships

Yuan NP, Teufel-Shone NI, Mayer B, Clichee D. Guidelines for Community Advisory Boards to Engage in Research. Center for Indigenous Environmental Health Research. Tucson, AZ; 2019.

Developing and Sustaining Community-Based Participatory Research Partnerships: A skill building curriculum. Developing and Sustaining Community-Based Participatory Research Partnerships: A Skill Building Curriculum. Accessed September 5, 2023. https://www.cbprcurriculum.info/.

Kubicek K, Robles, M. Resource for Integrating Community Voices into a Research Study: Community Advisory Board Toolkit. Southern California Clinical and Translational Science Institute. Los Angeles, CA; 2016.

Prioritization

Guide to Prioritization Techniques. National Association of County & City Health Officials. Washington, DC. https://www.naccho.org/uploads/downloadable-resources/Gudie-to-Prioritization-Techniques.pdf

Popular Business Design Tools. Business Design Academy. April 8, 2022. Accessed September 5, 2023. https://academy.dotankdo.com/tools/.

Evaluation

Goodman, MS, Ackermann, N, Bowen, DJ, & Thompson, V. (2019). Content validation of a quantitative stakeholder engagement measure. Journal of community psychology, 47(8), 1937-1951.

Thompson VLS, Leahy N, Ackermann N, Bowen DJ, Goodman MS Community partners' responses to items assessing stakeholder engagement: Cognitive response testing in measure development. PLoS ONE. 2020 15(11): e0241839.
