# Supplementary material for: Community advisory boards as implementation strategies to center partner and patient voice in community health centers
Source: J Clin Transl Sci. 2024 Dec 18;9(1):e14. doi: 10.1017/cts.2024.679 (PMC11795859; doi:10.1017/cts.2024.679)
Supplement: Lee et al. supplementary material 2 — Lee et al. supplementary material [file S2059866124006794sup002.docx]

**I. BACKGROUND/PARTNERSHIPS (10 Minutes) **skip for CHC staff****

1. First, to help me understand who was involved with this CHC-based community advisory group [focused on COVID-19], will you please describe how you came to join?

*PROBES:*

- Who invited you to join?
- What drew you to join?
- What did you hope to accomplish?
- How did you think your motivations aligned with those of the CHC?
- Past experience working with the CHC?
- If government/CBO: Where do you work? What is your title within your organization?
- If patient/resident/caregiver role: What city/neighborhood do you live in?

**II. ENGAGEMENT (30 minutes)**

In [insert month], you answered a survey about your engagement in the CHC-based community advisory group. Today I’d like to discuss your experience in greater depth.

1. What do you see as the greatest strengths of the community advisory group?
   1. Probe on items rated high on survey
2. What where the greatest challenges the community advisory group faced? How were they overcome?
   1. Probe on items rated low on survey
3. Thinking about the community advisory group meetings, were there aspects of the meeting that worked well? Needed improvement?
4. How was the communication between meetings? What did it look like? Were there aspects of the communications that worked well? Needed improvement?
5. How did leadership follow through on group input?
6. This CHC-based community advisory group was designed to improve access COVID-19 testing in your community. What was one of the greatest accomplishments of the group? Any accomplishments that wouldn’t have been possible without the community advisory group?

**III. RECOMMENDATIONS (10 minutes)**

1. How do you think your experience on the CHC-based community advisory group could translate into community-based action for other health issues? What were some of the most important aspects of your experience that groups should use in the future?
2. If you could speak to a CHC leader who is about to initiate their first community advisory group, what advice would you give them?

**Potential probes:** Meeting structure? Group composition? Voices missing? How to recruit?
